# Heat Stress and the Bull Sperm Transcriptome: Molecular Mechanisms, Fertility, and Epigenetic Inheritance

**DOI:** 10.3390/ani16182953

**Published:** 2026-09-20

**Authors:** Daniela Franco da Silva, Fabíola F. Paula-Lopes, Ticiano Guimarães Leite, Sónia Macedo, Vera Pessoa, Rosa M. L. N. Pereira

**Affiliations:** 1 Department of Veterinary Medicine, EUVG—Vasco da Gama University School, 3020-210 Coimbra, Portugal; daniela.silva@euvg.pt (D.F.d.S.);; 2 CIVG–Vasco da Gama Research Centre, Department of Veterinary Sciences, EUVG—Vasco da Gama University School, 3020-210 Coimbra, Portugal; 3 Institute of Biosciences of Botucatu—Júlio de Mesquita Filho, São Paulo State University (UNESP), Botucatu 18610-034, SP, Brazil; 4 School of Agriculture, Polytechnic University of Santarém, 2001-904 Santarém, Portugal; 5 Instituto de Ciências Ambientais, Químicas e Farmacêuticas—Universidade Federal de São Paulo (UNIFESP), Diadema 09972-270, SP, Brazil; 6 Laboratory of Semen Biotechnology and Andrology, Department of Animal Reproduction, School of Veterinary Medicine and Animal Science, University of Sao Paulo (USP), Pirassununga 13635-900, SP, Brazil; 7 CIISA—Centre for Interdisciplinary Research in Animal Health, Faculty of Veterinary Medicine, University of Lisbon, 1649-004 Lisboa, Portugal; 8 AL4Animals—Associate Laboratory for Animal and Veterinary Sciences, Faculty of Veterinary Medicine, University of Lisbon, 1649-004 Lisboa, Portugal; 9 Biotechnology and Genetic Resources Unit, Instituto Nacional de Investigação Agrária e Veterinária (INIAV), 2005-424 Vale de Santarém, Portugal

**Keywords:** spermatogenesis, spermatozoa, non-coding RNAs, microRNAs, oxidative stress, thermotolerance, embryonic development, paternal programming

## Abstract

Heat stress (HS) occurs when the body’s physiological mechanisms for regulating body temperature are unable to cope with external conditions. Regarding male reproductive function, HS increases testicular temperature, leading to a reduction in sperm production and motility, and an increase in abnormal sperm, oxidative stress and DNA fragmentation. However, the molecular basis behind these induced HS changes has not been fully characterized. In bovine spermatozoa, post-transcriptional regulation of messenger RNA (mRNA) is activated during spermatogenesis, and mechanisms mediated by microRNAs (miRNAs) are involved in the control of gene expression throughout the germline differentiation. Furthermore, extracellular vesicles containing proteins, lipids, miRNAs, mRNAs, and long non-coding RNAs derived from seminal plasma and epididymis influence sperm motility, capacitation, and fertilization potential. Therefore, despite the fact that mature spermatozoa are in an inactive transcriptional state, these cells contain a complex population of RNAs playing a role in embryonic development, which can be exploited for research on and diagnosis of male infertility. This review aims to elucidate several aspects of bovine sperm physiology and molecular mechanisms. Particular attention is given to the cascade of functional, cellular, and molecular events triggered by elevated temperatures in mature spermatozoa to disclose strategies to mitigate HS effects and maintain bull fertility.

## Simple Summary

Heat stress (HS) is characterized by an increase in core body temperature due to the failure of thermoregulatory mechanisms to cope with external conditions. In males, HS leads to an increase in testicular temperature, reducing spermatozoa production and quality as well as the ability of the spermatozoa to fertilize the female gamete and develop into healthy embryos. The molecular mechanisms behind HS-induced changes are not yet fully understood. This review aims to elucidate several aspects of bovine sperm physiology and the associated molecular mechanisms. Particular attention is given to the cascade of functional, cellular, and molecular events triggered by elevated temperatures in mature spermatozoa with the aim of disclosing strategies to mitigate the effects of HS and maintain bull fertility.

## 1. Introduction

Our planet is experiencing global climate change. Over the past decade, Europe has been particularly affected by global warming, with the Iberian Peninsula as one of the regions most impacted by increasingly intense and prolonged seasonal heat waves [1,2,3]. The impacts of global warming include food and water insecurity, ocean acidification, the reduction of polar ice caps, rising sea levels, and the reduction and loss of biodiversity [3]. Furthermore, heat stress (HS) poses a threat to mammalian species, including wildlife and livestock animals, resulting in physiological consequences when these species are exposed to high environmental temperatures. Throughout this review, HS refers to the physiological condition that results from exposure of the animal to elevated environmental temperature, when thermoregulatory mechanisms are unable to maintain body temperature within the thermoneutral zone; it is therefore used when describing in vivo exposure of sires and its systemic and testicular consequences. Heat shock denotes the direct exposure of cells, and, in particular, of ejaculated spermatozoa, to elevated temperature under controlled in vitro conditions. Thermal stress is used as a general term encompassing both situations, when no distinction between in vivo and in vitro exposure is intended.

To understand the impact of climate change on cattle, their evolutionary adaptation to different thermal environments must be considered. The *Bos taurus taurus* and *Bos taurus indicus* subspecies evolved under different environmental conditions for hundreds of thousands of years, resulting in adaptation to different climates. During this time, *Bos taurus indicus* has undergone genetic adaptations, acquiring genes for thermotolerance [4]. However, different levels of thermotolerance can also be identified among the *Bos taurus taurus*, depending on their environment of origin [5,6]. Even so, global warming has a significant impact on the health of farm animals, particularly cattle. HS has been identified as the main factor responsible for decreased animal welfare, fertility and productivity [7,8].

Infertility and subfertility in bulls are major problems in livestock production, since they have a significant impact on the reproductive performance of the herd. Among the various factors that cause a decrease in bull reproductive performance, adverse environmental conditions are highlighted [1,9]. Adult Bos taurus taurus cattle are generally reported to maintain thermal comfort within a thermoneutral zone extending up to approximately 25 °C, whereas Bos taurus indicus cattle may remain physiologically stable at ambient temperatures approaching 28–30 °C, although these thresholds vary according to breed, production level, humidity, solar radiation, and acclimation [10,11]. Temperatures above these thresholds cause HS, leading to increased scrotal and testicular temperature with consequent testicular degeneration [10]. The deleterious effects of HS on spermatogenesis and bull fertility have been well characterized. HS increases sperm morphological abnormalities, oxidative stress and DNA fragmentation, while reducing embryo development and quality [12,13,14,15,16,17]. Moreover, Vanselow et al. [12] demonstrated that heat effects on semen quality parameters were transmitted to the next generation, probably via sperm small non-coding RNAs (sncRNA).

Indeed, recent studies have reported that excessive heat affects the expression of messenger RNA (mRNA) and microRNA (miRNA) in sperm, which have important roles in regulating male fertility [16,18,19]. Although the fully mature spermatozoon is a highly differentiated and functionally quiescent cell, there is evidence that part of the sperm DNA remains bound to the histones during the DNA compaction process, suggesting that the spermatozoon nucleus may be a more dynamic structure than previously thought [20,21]. On the other hand, during fertilization, the spermatozoon carries the paternal genome into the oocyte. However, evidence indicates that mammalian sperm cells contribute not only paternal DNA but also a variety of coding and non-coding RNAs. It is thought that these RNAs trigger cellular and molecular events during embryo development, which are currently under investigation [21,22].

Studies using knockout mouse models have revealed the role of miRNAs in spermatogenesis [23]. In bovine spermatozoa, post-transcriptional regulation of mRNA is activated during spermatogenesis, and mechanisms mediated by miRNAs are involved in the control of gene expression throughout the course of male germ cell differentiation [16,24]. Furthermore, extracellular vesicles (EV) from the seminal plasma and the epididymis, which contain proteins, lipids, miRNAs, mRNAs, and long non-coding RNAs, influence sperm motility, capacitation, and fertilization potential, pointing out a role for EVs as carriers of regulatory molecules acting in spermatozoa [25,26].

In addition, recent studies have identified a miRNA that represses the expression of the heat shock factor 2 (HSF2) during spermatogenesis. HSF2 is a transcription factor that influences a wide range of cellular processes, including fertility/infertility [27]. Furthermore, during the HS response, a few minutes after the temperature rise, heat shock factor 1 (HSF1) is activated and interacts with HSF2, enhancing the transactivation mediated by HSF1, resulting in an increase in the transcription of heat shock proteins (HSPs) [28]. Björk et al. [29] reported that miR-18 directly binds to the HSF2 during spermatogenesis in mice and that miRNA-18 is highly abundant in the testis, displaying distinct cell-type-specific expression during spermatogenesis. Conversely, the miRNA profiling of bovine sperm has identified >300 miRNAs [17]. Among these, three and seven miRNAs were exclusively identified in sperm cells exposed to 35 and 41 °C, respectively. Thus, the miRNA profile was altered by elevated temperatures perturbing the physiology of mature sperm cells. This led to a lower ability to fertilize the oocytes and subsequent preimplantation embryo development [17]. However, the molecular mechanisms behind these HS-induced changes remain elusive.

The aim of this review is to elucidate several aspects of sperm physiology and to highlight molecular mechanisms in bovine spermatozoa that are related to the physiological response to HS. It draws on studies published between 1963 and 2026, which were identified through searches in scientific databases. Particular attention is given to the cascade of functional, cellular, and molecular events triggered by elevated temperatures in the mature spermatozoa, with the purpose of disclosing strategies to mitigate the effects of HS and maintain bull fertility.

## 2. Spermatogenesis in Bulls

Spermatogenesis is the highly orchestrated process of proliferation, meiosis, and cellular differentiation through which diploid spermatogonia are transformed into haploid spermatozoa within the seminiferous tubules of the testis [30,31]. In the bull, spermatogenesis is conventionally divided into three sequential phases: a proliferative phase, in which spermatogonia undergo successive mitotic divisions; a meiotic phase, in which spermatocytes generate haploid spermatids; and spermiogenesis, during which spermatids are remodeled into mature spermatozoa [32,33]. The fidelity of this sequence is essential for male fertility, and disruption due to genetic, environmental, or stress factors directly reflects on the quantity and quality of the ejaculate.

The foundations of spermatogenesis are laid during embryonic development. Primordial germ cells (PGCs) arise outside the gonad and migrate along the hindgut to colonize the genital ridges. Once enclosed within the developing seminiferous cords, PGCs differentiate into gonocytes, also termed prospermatogonia. These cells remain largely quiescent until the neonatal period, when they give rise to the spermatogonial population, including the spermatogonial stem cells that sustain sperm production throughout the reproductive life of the bull [34].

During spermatocytogenesis, bovine spermatogonia proliferate through a defined sequence of mitotic divisions, comprising A0, A1, A2 and A3 spermatogonia followed by intermediate and type B spermatogonia, ultimately giving rise to primary spermatocytes [32,35]. These enter meiosis to produce secondary spermatocytes and, in turn, haploid round spermatids, which are subsequently reshaped into spermatozoa during spermiogenesis. In the bull, the entire process spans approximately 61 days, comprising about 21 days of mitotic proliferation, 23 days of meiosis, and 17 days of spermiogenesis [31,32,36]. This estimate is species-specific and relatively constant between individuals [30,32].

A hallmark of germ cell development is incomplete cytokinesis: daughter cells remain connected by stable intercellular bridges that allow the exchange of cytoplasm and gene products and thereby synchronize the differentiation of germ cell clones [37]. Spermiogenesis entails extensive morphological remodeling, including acrosome biogenesis, flagellar assembly, nuclear elongation, and chromatin condensation. During this phase, most histones are replaced by protamines, enabling the dense packaging of paternal DNA and conferring the genomic protection required for fertilizing competence [23]. Still, spermatozoa leaving the testis are not functionally competent. Post-testicular maturation in the epididymis is needed to confer progressive motility and fertilizing ability through biochemical and membrane modifications driven by the distinctive luminal environment and secretory activity of the epididymal epithelium [38].

At the molecular level, spermatogenesis is governed by tightly coordinated transcriptional and post-transcriptional programs. Transcription is progressively silenced as chromatin condenses during late spermiogenesis. At this point, post-transcriptional control by sncRNAs assumes a central role [23,39]. These regulatory molecules, namely miRNAs, endogenous small interfering RNAs (endo-siRNAs), and PIWI-interacting RNAs (piRNAs), contribute to germ cell differentiation, transposon silencing, and genome integrity [23]. In bovine spermatogenesis, miRNAs have emerged as key modulators of gene expression and as candidate markers of fertility [40].

Heat shock transcription factors also participate in the regulation of spermatogenesis. HSF2 is expressed in meiotic and post-meiotic germ cells. Genome-wide analyses demonstrated HSF2 localization to the male-specific region of the Y chromosome, where it regulates resident multicopy genes, including Ssty (Spermiogenesis specific transcript on the Y), a multicopy Y-linked gene encoding the testis-specific SSTY1 and SSTY2 proteins involved in the late stages of spermiogenesis and required for normal sperm formation [41]. Deletions affecting this region produce sperm head abnormalities, impaired fertilization and, in the most extensive cases, sterility in animal models [41,42]. More recent genomic and transcriptomic analyses have refined the catalog of Y-linked genes essential for spermatogenesis, reinforcing the contribution of this chromosome to male fertility [33,43].

Germ cell development does not proceed in isolation. Sertoli cells provide the structural, nutritional, and regulatory support that sustains the seminiferous epithelium, and several Sertoli-expressed transcription factors, among them WT1, SOX8, and RHOX5, are indispensable for epithelial maintenance and the orderly progression of germ cell differentiation [44]. Other regulators act beyond the testis: FOXI1, for instance, governs aspects of epididymal function that contribute to the final stages of sperm maturation [45], and continuing work is further clarifying the secretory role of Sertoli cells during spermatogenesis [46]. Single-cell transcriptomic atlas of the bovine testis has recently resolved this cellular heterogeneity in detail, mapping the developmental trajectory from spermatogonia to spermatozoa and refining stage-specific markers for germ and somatic cell populations alike [47].

The mature spermatozoon is a highly polarized cell whose architecture is precisely adapted to fertilization, comprising a head that houses the condensed haploid nucleus and the acrosome, a mitochondria-rich midpiece that supplies energy, and a flagellum that drives motility [35]. Its plasma membrane is organized into discrete functional domains responsible for zona pellucida binding, the acrosome reaction, and fusion with the oocyte, domains that are established during spermiogenesis and refined during epididymal maturation [48,49]. This structural and molecular specialization, together with the post-transcriptional regulatory machinery outlined above, constitutes the cellular substrate upon which HS acts; the functional, chromatin, and small-RNA alterations elicited by elevated temperature, and their possible mediation by extracellular vesicles, are addressed in the sections that follow.

## 3. Heat Stress in Sires: Physiological and Testicular Responses

Climate change is among the foremost challenges facing livestock production in the twenty-first century. According to the Intergovernmental Panel on Climate Change (IPCC), the global surface temperature in 2011–2020 was approximately 1.1 °C higher than in the 1850–1900 pre-industrial baseline and, depending on the emissions scenario, is projected to rise by a further ~1.4 °C (very low emissions) to ~4.4 °C (very high emissions) by 2081–2100, with the high-emissions pathway reaching 3.3–5.7 °C [50,51]. This warming trend is particularly deleterious for cattle production in tropical and subtropical regions, where animals already operate close to, or beyond, the upper limits of the thermoneutral zone [19].

HS occurs when environmental conditions exceed the animal’s capacity to dissipate heat, resulting in hyperthermia and disruption of physiological, metabolic, cellular, and molecular homeostasis [52]. In regions characterized by persistently high ambient temperatures, HS compromises cattle productivity and fertility [9]. Even seasonal increases in environmental temperature can impair bull reproductive performance, with semen quality declining during warmer months [53,54].

Normal spermatogenesis in mammals requires testicular temperature to be maintained approximately 4–5 °C below core body temperature [13,55]. This thermoregulatory capacity is supported by a countercurrent heat-exchange system involving the testicular vascular cone and pampiniform plexus, through which arterial blood is cooled before reaching the testis [56]. When elevated ambient temperature exceeds the capacity of this system, testicular temperature increases, potentially resulting in testicular degeneration and impaired spermatogenesis [57]. Because developing germ cells differ in their susceptibility to thermal injury, the consequences of HS are delayed rather than immediate. Defective spermatozoa emerge in a predictable, stage-dependent sequence from approximately 11 days after heat insult, while recovery of semen quality generally requires completion of spermatogenesis and epididymal transit, taking approximately 30–40 days after a transient insult and considerably longer after prolonged HS [58].

Thermotolerance varies substantially among cattle subspecies and breeds. Zebu cattle (*Bos taurus indicus)* are generally better adapted to tropical environments than taurine cattle (Bos taurus taurus), owing to genotypic and phenotypic characteristics that promote heat dissipation [58,59]. These include a greater surface area to body mass ratio, a shorter and sleeker hair coat, and a greater density of sweat glands [4,9]. Conversely, European taurine breeds are generally more susceptible to HS under tropical conditions, partly because of less efficient heat-dissipation mechanisms, which can result in increased testicular temperature and impaired spermatogenesis [10]. Importantly, thermotolerance is not determined by subspecies alone. Substantial inter-individual variation exists within breeds, with some bulls maintaining semen quality during thermal challenge whereas others exhibit pronounced deterioration [54]. Certain locally adapted taurine populations have also developed marked thermotolerance through natural selection in hot environments. One notable example is the SLICK phenotype, associated with dominant mutations in the prolactin receptor (PRLR) gene, which produce a short, sleek hair coat that enhances heat dissipation and has been successfully introduced into Holstein cattle [60,61]. These observations highlight the genetic basis of thermotolerance and suggest that molecular mechanisms may contribute to individual differences in the response to thermal stress. In particular, EVs and their miRNA cargo have emerged as potential mediators of cellular responses to HS and as candidate molecular biomarkers of thermotolerance [16,19]. These molecular mechanisms are considered further in the following sections.

## 4. Heat Stress and Bull Reproductive Performance

In vivo exposure of sires to HS raises scrotal and testicular temperature, reducing semen quality, altering sperm morphology and motility, and compromising the fertilizing capacity of spermatozoa, ultimately jeopardizing subsequent embryo development [58,62]. The scrotal insulation model has been widely used to reproduce these effects experimentally and has consistently revealed breed-dependent responses. A clear illustration was provided by the comparison between Holstein and Curraleiro bulls subjected to 48 h of scrotal insulation [62]. In the post-insulation period, progressive sperm motility declined considerably more in Holstein bulls (50%) than in Curraleiro bulls (25%). In addition, the parallel decline in the subjective sperm vigor score (scale 0–5) was likewise greater in Holstein (47.8%) than in Curraleiro (15.2%) bulls. Curraleiro bulls also recovered their pre-insulation motility much earlier (by day 37) than Holstein bulls (by day 51). In Holstein bulls, the proportion of morphologically abnormal spermatozoa exceeded 30% between days 16 and 44 post-insulation, reaching a peak of 41.3 ± 5.9% on day 30. In the same breed, the significant increase in proximal cytoplasmic droplets from day 23 onwards was interpreted as evidence of injury to spermatids or spermatocytes. By contrast, Curraleiro bulls exhibited a milder response to scrotal insulation, consistent with their greater thermotolerance [62]. Importantly, the Curraleiro is not a zebu breed but a naturalized taurine breed (*Bos taurus taurus*) of Iberian origin, whose thermotolerance results from several centuries of natural adaptation to the semi-arid Brazilian environment. This contrast is therefore particularly informative as it demonstrates that pronounced thermotolerance can emerge within *Bos taurus taurus* through environmental selection, independently of indicine introgression, reinforcing the view that thermotolerance is a heritable trait amenable to molecular characterization and genetic selection.

A detailed demonstration of the temporal effects of scrotal HS comes from a study in Nellore (*Bos taurus indicus*) and Canchim (*Bos taurus indicus × Bos taurus crossbred*) bulls subjected to whole-scrotum insulation for 96 h [58]. In this study, the effects of scrotal insulation were initially evaluated through changes on fresh semen quality. The ejaculates collected from the bulls were subsequently cryopreserved, thawed, and used for in vitro fertilization. This distinction is important because the cellular consequences of HS in spermatozoa exposed to scrotal insulation in vivo should be interpreted separately from those occurring after cryopreservation and thawing, as the latter process itself imposes cryogenic and osmotic stress that may further affect sperm function. In the Nellore bulls, sperm motility in fresh semen declined significantly on days 11 and 14, while specific morphological defects emerged in an orderly and predictable sequence: loose sperm heads on day 11, midpiece defects between days 11 and 14, proximal cytoplasmic droplets between days 14 and 27, sperm vacuoles between days 18 and 21, and acrosome defects between days 27 and 31. This temporal pattern reflects the differential susceptibility of successive germ-cell populations to scrotal heat stress, particularly spermatids and spermatocytes, and may also involve alterations in epididymal maturation.

Beyond morphology and motility, HS impairs the functional competence of spermatozoa and the developmental potential of the resulting embryos. When assessed by in vitro fertilization, cleavage rates of heat-stressed semen remained comparable to those of controls until day 14 post-insulation but declined significantly by day 21, while blastocyst rates fell from day 14 onwards, in parallel with increasing sperm head and chromatin defects [14]. These observations indicate that HS affects not only the structural and kinematic attributes of spermatozoa but also their fertilizing and embryotrophic ability.

Field observations corroborate the experimental findings, as bulls exhibit higher percentages of sperm head abnormalities and proximal droplets during the summer [63]. Among the mitigation strategies investigated, dietary supplementation with omega-3 fatty acids has shown promise in protecting fresh semen quality, although no benefit was evident in frozen-thawed samples [64]; antioxidant supplementation has likewise been proposed to counteract the oxidative component of heat-induced sperm damage [65].

Recent research has extended this picture beyond morphology and motility to molecular and epigenetic alterations. HS modifies sperm chromatin condensation [66], DNA methylation patterns, and miRNA expression profiles [19,67]. Notably, transient scrotal HS alters the miRNA cargo of both spermatozoa and seminal plasma EVs in bulls [16], with concurrent changes in testicular miRNA profile [19]. These findings raise the possibility that EVs and their miRNA cargo act as molecular mediators of the spermatozoa and testicular response to HS, acting as biomarkers of thermotolerance. Combined with the substantial inter-individual variation observed among bulls [54], this opens a clear avenue for the molecular characterization and genetic selection of thermotolerant sires as an adaptation strategy in the face of climate change [57]–the rationale underpinning the present review of miRNAs as modulators of the molecular response to HS in bovine testis and spermatozoa.

## 5. Direct Heat Shock of Bovine Spermatozoa: Cellular and Molecular Responses

Several in vitro studies have consistently demonstrated that heat shock exerts deleterious effects on bovine sperm function in a temperature- and time-dependent manner. Exposure of spermatozoa to temperatures ranging from 41 to 43 °C significantly impairs total and progressive motility, plasma membrane integrity, mitochondrial membrane potential, and fertilizing ability, whereas incubation at physiological temperature (38.5–39 °C) preserves sperm functionality [66,67,68,69,70,71]. Collectively, these findings indicate that temperatures above the physiological range rapidly compromise sperm viability and function, with the magnitude of damage increasing with exposure time.

Among the cellular structures affected by HS, the plasma membrane and mitochondria appear to be particularly susceptible. Maya-Soriano et al. [69] demonstrated that incubation at 41.5 °C significantly reduced sperm motility and plasma membrane integrity while increasing acrosomal alterations, although sperm morphology remained unaffected. Similarly, Rahman et al. [68] reported marked reductions in sperm motility, plasma membrane integrity, and mitochondrial membrane potential following incubation at 41 °C, indicating that mitochondrial dysfunction is an early event in heat-induced sperm damage. Because mitochondria provide the ATP required for flagellar movement, impairment of mitochondrial activity likely contributes directly to the decline in sperm motility consistently observed across studies. The detrimental effects of HS extend beyond sperm quality and directly compromise reproductive performance. Incubation of bovine spermatozoa at elevated temperatures reduces fertilization rates, embryo cleavage, and blastocyst development while increasing apoptosis in preimplantation embryos [68,72]. Interestingly, although Rahman et al. [68] demonstrated that inhibition of the p38 MAPK (Mitogen-activated protein kinase) pathway reduced MAPK14 activity, this intervention failed to restore fertilization or embryonic development, suggesting that heat-induced sperm damage involves multiple interconnected molecular pathways rather than a single signaling mechanism.

Recent studies have provided further mechanistic insight into the cellular responses triggered by heat shock. Silva et al. [17] demonstrated that exposure of frozen-thawed bovine spermatozoa to 41 °C increased reactive oxygen species (ROS) production and caspase activity while reducing mitochondrial function and sperm motility. Importantly, heat shock also altered the sperm miRNA profile, indicating that thermal stress affects not only sperm physiology but also its epigenetic cargo, which may contribute to impaired fertilization and early embryonic development. HS has been shown to disrupt sperm structures and molecular components involved in fertilization [73,74]. Heat shock alters the organization of cytoskeletal proteins, including actin and tubulin, which play essential roles in acrosomal exocytosis [74]. These alterations may promote premature acrosomal loss, thereby reducing the ability of spermatozoa to successfully penetrate the oocyte [19]. Independently of HS, proteomic studies of bovine spermatozoa have identified proteins associated with key reproductive functions, including capacitation, sperm–oocyte interaction, and fertilizing competence, supporting the potential of proteomic approaches for identifying molecular biomarkers of bull fertility [75]. Mechanistically, Santos et al. [73] demonstrated that exposure to 41 °C alters the localization of Ca^2+^/calmodulin-dependent protein kinase II (CaMKII), accelerating changes in acrosomal membrane integrity and promoting redistribution of phosphorylated CaMKII. Together, these findings indicate that HS can impair sperm fertilizing competence through coordinated disruption of structural integrity and intracellular signaling pathways. More broadly, omics approaches may help identify molecular biomarkers of sperm function and bull fertility and improve the assessment of reproductive competence [76].

Given the multifactorial nature of heat-induced sperm damage, several strategies have been investigated to improve sperm resilience. Weldon & Fair [77] demonstrated that ectoine, a compatible solute naturally produced by extremophilic bacteria, preserved sperm motility and viability under thermal stress (39–42 °C), with the highest concentration producing approximately a twofold increase in sperm viability compared with untreated controls. These findings identify ectoine as a promising cryoprotective and thermoprotective molecule for bovine sperm preservation.

Another emerging strategy involves the use of seminal plasma-derived EVs, which mediate intercellular communication through the transfer of proteins, lipids, and regulatory RNAs [78,79,80,81,82]. EVs are enriched in HSPs, tetraspanins, integrins, sphingolipids, cholesterol, and diverse sncRNAs, enabling them to modulate sperm physiology and cellular stress responses. Consistent with this role, Lange-Consiglio et al. [78] demonstrated that supplementation of spermatozoa from low-fertility bulls with EVs isolated from the seminal plasma of a fertile bull approximately doubled day-7 blastocyst production following in vitro fertilization. Similarly, Pal et al. [83] reported that EVs from high-fertility Sahiwal bulls were enriched in fertility-associated proteins, including SP10 (Sperm Intra-Acrosomal Protein/Antigen SP-10), ADAM7 (A Disintegrin and Metalloproteinase 7), and SPAM1 (Sperm Adhesion Molecule 1/Hyaluronidase PH-20), and improved sperm function by reducing ROS production while modulating capacitation and acrosomal responsiveness. Although the application of EVs in assisted reproduction remains experimental, these findings suggest that EV-mediated delivery of bioactive molecules represents a promising strategy to enhance sperm quality and potentially mitigate the detrimental effects of HS.

Overall, the available evidence consistently demonstrates that heat shock impairs bovine sperm function through multiple interconnected mechanisms, including mitochondrial dysfunction, oxidative stress, disruption of membrane integrity, cytoskeletal remodeling, altered intracellular signaling, and changes in the sperm epigenetic cargo. These alterations ultimately compromise fertilization and early embryonic development, highlighting the importance of developing therapeutic strategies capable of preserving sperm functionality under conditions of HS.

## 6. The Sperm RNA

Mature spermatozoa are highly specialized cells that not only deliver the paternal genome to the oocyte but also carry a complex repertoire of RNA molecules and epigenetic information capable of influencing fertilization and early embryonic development [84,85]. Transcriptomic studies have revealed that mature spermatozoa contain a diverse population of coding and non-coding RNAs, including mRNAs, miRNAs, piRNAs, transfer RNA-derived small RNAs (tsRNAs), circular RNAs (circRNAs), and long non-coding RNAs (lncRNAs) [86,87,88]. Although each RNA class contributes to sperm function, accumulating evidence indicates that only a subset exerts direct regulatory effects following fertilization.

Among sperm-borne miRNAs, miR-34c is the best characterized, having been implicated in the first embryonic cleavage and the regulation of early embryonic development after delivery to the oocyte [89]. Members of the miR-449 family also contribute to mice spermatogenesis and early embryogenesis through partially overlapping regulatory pathways [90,91]. More recently, tsRNAs have emerged as major mediators of paternal epigenetic inheritance. These small RNAs are acquired during epididymal maturation via epididymosomes and modulate gene expression, embryo development, and offspring metabolic phenotypes [92,93]. In contrast, piRNAs primarily safeguard genome integrity during spermatogenesis by suppressing transposable elements [94], whereas circRNAs and lncRNAs are thought to regulate gene expression through mechanisms such as acting as miRNA sponges, RNA scaffolds, and modulators of transcription and chromatin organization, although their post-fertilization functions remain less well defined [95]. Instead of representing passive remnants of spermatogenesis, many of these RNA molecules are now recognized as functional regulators of fertilization, early embryonic development, and paternal epigenetic inheritance [86,96,97]. Although the etiology of male infertility remains multifactorial and, in many cases, poorly understood, increasing evidence suggests that environmental and systemic factors can impair spermatogenesis, compromise sperm function, and ultimately reduce male fertility [98,99,100]. Furthermore, physiological increases in scrotal and testicular temperature impair spermatogenesis and compromise semen quality [101]. At the cellular level, prolonged heat exposure and post-ejaculatory HS disrupt sperm ultrastructure, increase oxidative damage, and reduce subsequent embryonic developmental competence, including blastocyst formation [17,72]. Likewise, exposure to toxic chemical compounds elevates intracellular ROS levels, leading to impaired sperm motility, disrupted calcium homeostasis, and reduced fertilization capacity [102,103]. Accordingly, several exogenous antioxidants have recently been investigated as potential therapeutic strategies. Among them, Lin et al. [104] demonstrated that curcumin supplementation effectively alleviates oxidative stress-induced damage, restoring sperm motility and improving fertilization rates in bovine models. Collectively, these observations indicate that diverse environmental stressors converge on common molecular mechanisms involving oxidative stress and mitochondrial dysfunction. Contemporary evidence further demonstrates that pesticides, herbicides, heavy metals, microplastics, and endocrine-disrupting compounds exacerbate these processes by promoting oxidative DNA damage and disrupting hypothalamic-pituitary-gonadal axis signaling [105,106,107]. Together with increasing HS associated with climate change, these environmental exposures are considered major contributors to declining semen quality and may induce heritable epigenetic alterations across generations [108].

Male fertility depends on the production of morphologically normal, motile, and functionally competent spermatozoa [109,110]. Consequently, sperm quality assessment extends beyond conventional parameters such as concentration, motility, morphology, membrane integrity, and vigor to include the ability of spermatozoa to complete the sequential events required for successful fertilization. These include migration through the female reproductive tract, capacitation, acrosome reaction, zona pellucida penetration, oocyte activation, and the maintenance of genomic and transcriptomic integrity required to sustain normal embryonic development and healthy offspring [111,112,113].

During spermatogenesis, chromatin undergoes extensive remodeling through the replacement of histones with protamines, resulting in progressive transcriptional silencing and the elimination of most cytoplasmic content by Sertoli cells [114,115,116]. Mature spermatozoa were therefore long regarded as transcriptionally and translationally inert cells. However, decades of experimental evidence have progressively challenged this paradigm. Early radiolabeling studies demonstrated RNA and protein precursor incorporation in mammalian spermatozoa [117] while subsequent investigations established that mature sperm retain limited translational activity mediated by mitochondrial, rather than cytoplasmic ribosomes [118,119,120]. This concept was further strengthened by Gur and Breitbart [121] showing that protein synthesis during capacitation is selectively inhibited by mitochondrial translational inhibitors. Collectively, these findings established that mature spermatozoa retain a restricted but functionally relevant metabolic capacity despite the absence of nuclear transcription.

**Figure 1:**
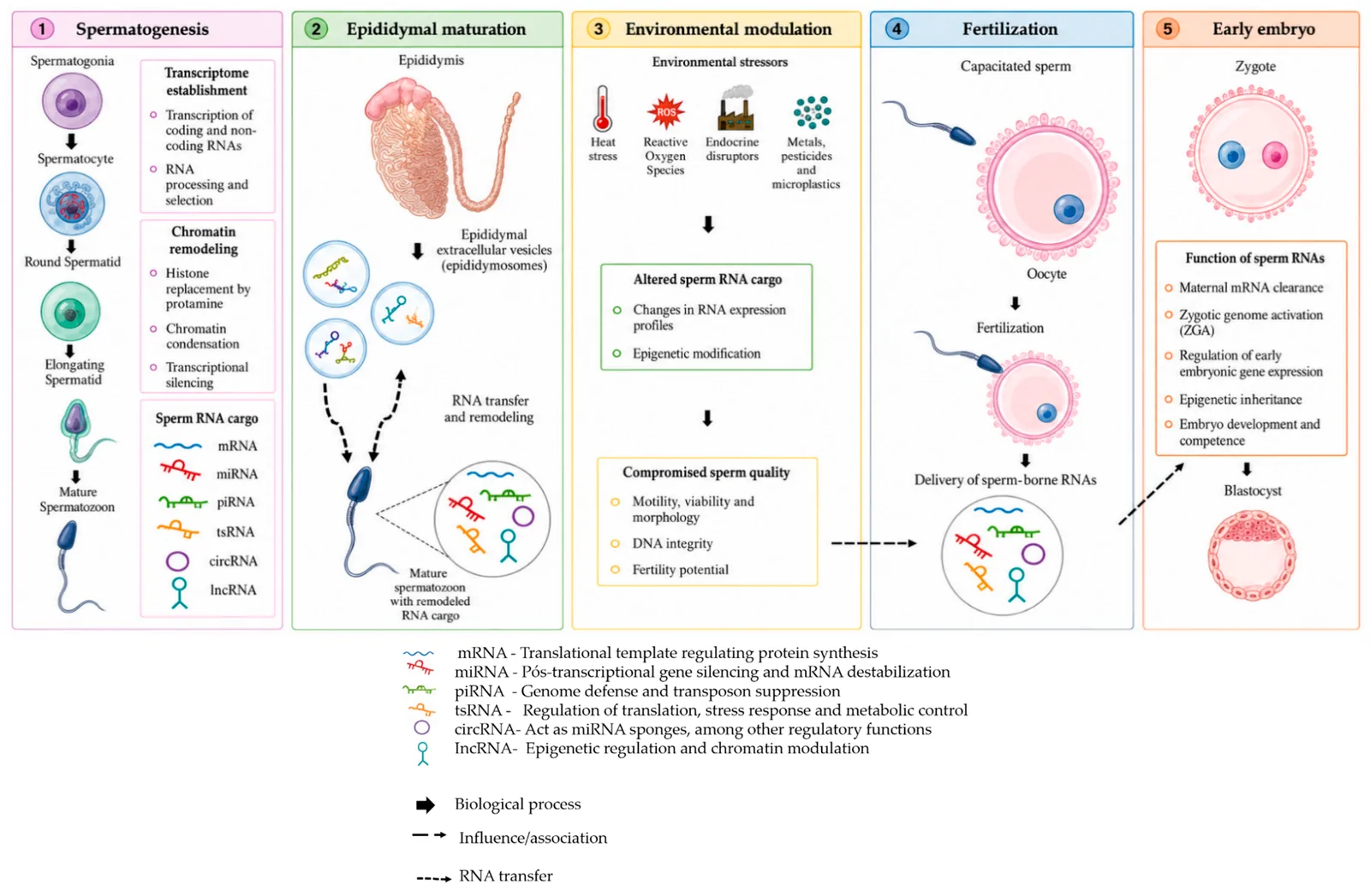
Overview of sperm RNA biogenesis, epididymal remodeling, and post-fertilization function.

Although nuclear transcription is largely absent in mature spermatozoa, the RNA cargo is now recognized as a molecular archive of spermatogenesis and epididymal maturation [120,122]. Sperm RNA abundance and composition reflect germ-cell differentiation and sperm quality, making these transcripts attractive biomarkers of male fertility [123]. Accumulated evidence indicates that the sperm transcriptome undergoes further remodeling during epididymal transit through the selective transfer of coding and non-coding RNAs by epididymosomes [124]. Following fertilization, these paternal RNAs are delivered to the oocyte, participating in maternal transcript clearance, zygotic genome activation, early embryonic gene regulation, and epigenetic inheritance [124,125,126,127]. Despite substantial advances in transcriptomic profiling, the precise biological functions of many sperm RNAs remain incompletely understood, particularly regarding the individual contributions to fertilization, embryo development, and transgenerational inheritance. An overview of sperm transcriptome establishment, chromatin remodeling, epididymal maturation, and post-fertilization functions is presented in Figure 1.

This paradigm shift has transformed the traditional view of spermatozoa from passive carriers of paternal DNA into active regulators of early embryonic development [128]. Accumulating evidence indicates that sperm-borne RNAs modulate maternal mRNA degradation, facilitate zygotic genome activation, and influence embryonic developmental competence through epigenetic mechanisms [85,122,129]. In addition to the regulatory RNA cargo, spermatozoa transmit a highly organized chromatin landscape in which retained nucleosomes are preferentially enriched at developmental genes and regulatory regions carrying histone modifications associated with transcriptional activation or repression [130,131,132]. Together, these epigenetic layers provide a mechanistic framework linking paternal environmental exposures with early embryonic gene regulation.

Advances in transcriptomic technologies, from microarray analysis and bulk RNA sequencing to single-cell RNA sequencing and spatial transcriptomics, have enabled increasingly comprehensive characterization of the sperm transcriptome and revealed the dynamic transcriptomic remodeling that occurs throughout spermatogenesis [133,134,135,136,137,138]. Instead of representing random remnants of spermatogenesis, the selective retention of specific RNA populations is increasingly recognized as a tightly regulated process with important functional implications for fertilization, early embryonic development, and paternal epigenetic inheritance.

## 7. miRNAs in Spermatozoa

miRNAs are ~22-nucleotide non-coding RNAs that regulate gene expression primarily through sequence-specific binding to target mRNAs, promoting transcript degradation or translational repression [139]. miRNAs are generated through sequential processing of primary transcripts by the Drosha-DGCR8 complex in the nucleus and Dicer in the cytoplasm before being incorporated into the RNA-induced silencing complex [140,141,142,143,144,145]. In addition to these canonical post-transcriptional functions, miRNAs can also modulate transcriptional activity and chromatin organization, expanding the regulatory influence over cellular gene expression [23]. The historical milestones underlying the discovery and functional characterization of miRNAs are summarized in Table 1.

It has been shown that miRNAs are indispensable regulators of spermatogenesis, orchestrating germ cell proliferation, differentiation, meiosis, and survival through highly coordinated, stage-specific expression patterns [147,150,151,152]. Distinct miRNA signatures have been identified in Sertoli cells, spermatogonia, spermatocytes, spermatids, and mature spermatozoa, highlighting the precise temporal and cellular regulation required for normal germ cell development [153,154,155]. In cattle, high-throughput small RNA sequencing demonstrated that haploid germ cells constitute the primary source of sperm-associated miRNAs, although the small RNA repertoire undergoes substantial remodeling during epididymal maturation before fertilization [154,156,157,158]. Several evolutionarily conserved miRNAs, including members of the miR-34 and miR-18 families, have emerged as key regulators of germ-cell differentiation by controlling genes involved in meiosis, spermiogenesis, and cellular stress responses [29,146,159].

Despite the absence of nuclear transcription, mature spermatozoa retain a stable and highly selective population of miRNAs that is delivered to the oocyte at fertilization [148,160]. Increasing evidence indicates that these paternal miRNAs participate in the regulation of maternal transcript turnover, zygotic genome activation, and early embryonic development, supporting an important role in paternal epigenetic inheritance [149,160]. Consistent with these functions, alterations in sperm and seminal plasma miRNA profiles have been associated with impaired fertility in both humans and livestock, highlighting their potential as minimally invasive biomarkers of male reproductive performance [92,148,157,161]. In humans, distinct miRNA signatures discriminate patients with asthenozoospermia and non-obstructive azoospermia, whereas in cattle, sperm miRNA profiling identifies subfertile bulls that cannot be reliably distinguished using conventional semen quality assessments [156,162]. Moreover, differentially expressed miRNAs are linked to pathways governing sperm structure, fertilization, embryonic development, and placental function, reinforcing their value as functional biomarkers of reproductive success [129,163]. However, the biological relevance of sperm miRNAs extends beyond their association with fertility outcomes, as several studies have provided functional evidence for their involvement in stress responses, sperm function, and early embryonic development. Sperm-borne miRNAs have therefore emerged as important regulators of male reproductive function and early embryonic development, with evidence from both mechanistic studies and fertility-associated investigations. The studies summarized in Table 2 illustrate the diverse roles of bovine sperm miRNAs in sperm function, fertility, stress responses, and early embryonic development. For instance, in a heat shock bovine model, miR-181d was enriched in spermatozoa exposed to 41 °C and associated with increased oxidative stress and reduced sperm quality, suggesting its potential role as a candidate molecular marker of heat-induced sperm stress [17]. In addition, functional studies demonstrated that sperm-derived miR-202 regulates the first embryonic cleavage by directly targeting SEPT7, promoting cytoskeletal remodeling through α-tubulin stabilization. Restoration of miR-202 levels or suppression of SEPT7 expression rescued cleavage defects in somatic cell nuclear transfer (SCNT)-derived embryos, providing direct evidence that sperm miRNAs can actively influence early embryonic development rather than simply serving as indicators of sperm quality [164].

High-throughput sequencing further indicates that male fertility relies on complex, coordinated miRNA networks rather than individual markers. Comparing bulls with similar conventional semen parameters but divergent conception rates, Werry et al. [156] identified distinct sperm miRNA profiles and altered correlation patterns. The abundance of specific miRNAs, including miR-34b-3p, miR-100-5p, miR-191-5p, miR-30d-5p, and miR-21-5p, together with pathway analyses linking these molecules to spermatogenesis and embryo development, supports the involvement of coordinated miRNA networks in sperm fertilizing capacity. Consistent with this concept, subsequent studies have identified specific sperm miRNAs associated with semen quality, cryotolerance, and reproductive performance. miR-31-5p has been proposed as a candidate marker of sperm freezability, as higher abundance in fresh semen is associated with improved post-thaw motility, DNA integrity, mitochondrial function, and reduced oxidative damage [165,166]. Similarly, bta-miR-138 is enriched in cryopreserved spermatozoa from high-fertility bulls and correlates negatively with oxygen consumption, suggesting improved metabolic efficiency. Additional miRNAs, including bta-miR-19b, bta-miR-26a, and bta-miR-7, are associated with post-thaw sperm functionality and kinematic parameters [167]. Furthermore, miR-10a-5p and miR-9-5p discriminate between bulls with different fertility levels, whereas miR-34c and miR-342 have been associated with non-return rate, reinforcing their potential as predictors of reproductive performance [168].

Collectively, these findings highlight the emerging dual function of sperm miRNAs as active regulators of paternal reproductive contribution and as molecular signatures associated with male fertility. Although mechanistic studies provide evidence that specific miRNAs directly influence sperm function and early embryonic development, fertility-associated studies indicate that integrated miRNA profiles may offer greater predictive value for reproductive performance than individual markers. Importantly, the sperm RNA landscape may be shaped by factors beyond thermal stress. Age-related developmental processes, including sexual maturation, as well as paternal metabolic and environmental conditions, may contribute to variation in sperm-borne small non-coding RNAs and the broader sperm epigenetic landscape [127,169,170,171].

**Table 2:** Bovine studies identifying sperm or reproductive tract associated miRNAs and their major molecular findings.

Study	Breed	Experimental Conditions	Evaluated Parameters	Major Altered RNAs/Molecular Findings	Current Relevance
Alves et al. (2021) [16]	Nellore	Transient scrotal heat stress; scrotal bags for 96 h; sperm and seminal plasma sEVs evaluated after heat exposure	Sperm and sEV miRNA profiles	21 sperm miRNAs altered (18 downregulated, 3 upregulated); several sEV miRNAs were also reduced. Altered miRNAs included miR-9-5p, miR-15a, miR-18a, miR-20b, miR-30a/b/d/e, miR-34b/c, miR-106b, miR-126-5p, miR-146a, miR-191, miR-192, miR-200b, miR-335 and miR-449a	Provides direct evidence that transient testicular heat stress modifies the bovine sperm miRNA cargo, supporting miRNAs as potential mediators of heat-stress effects on sperm function and fertility
Da Silva et al. (2022) [17]	Holstein-Friesian	Frozen-thawed sperm incubated at 35, 38.5 or 41 °C for 4 h to model heat shock.	Sperm motility, mitochondrial activity, ROS, caspase activity, fertilization and embryo development; sperm miRNA profiling	>300 miRNAs identified; 3 miRNAs were exclusive to 35 °C and 7 to 41 °C. miR-181d was enriched at higher temperatures. Heat shock also impaired sperm function and subsequent embryo development	Strong evidence linking thermal exposure to coordinated alterations in sperm physiology, miRNA composition and reproductive competence
Sellem et al. (2020) [96]	Holstein- Friesian, Montbéliarde, Normande, Charolais, Abondance, Belgian Blue	Comparative profiling of cryopreserved ejaculated sperm from 40 bulls representing six breeds	Small non-coding RNA sequencing and breed comparison	2723 unique miRNAs detected (701 known and 2022 predicted); 294 known miRNAs were differentially expressed among breeds. miRNAs represented ~20% of sperm sncRNAs	Establishes substantial breed-associated diversity in the bovine sperm miRNA repertoire, an important consideration when interpreting molecular biomarkers across populations
Sellem et al. (2021) [92]	Holstein- Friesian	Small-RNA profiling across the male reproductive tract: testicular parenchyma, epididymal caput, corpus, cauda and ejaculated sperm	Dynamics of sperm sncRNAs and miRNA abundance during maturation	The miRNA fraction increased progressively along the reproductive tract, from ~1% in testicular parenchyma to ~38% in ejaculated sperm. Target enrichment involved cell-cycle/stress pathways early and embryonic morphogenesis in ejaculated sperm	Supports progressive acquisition and remodeling of the sperm miRNA cargo during epididymal maturation, relevant to sperm function and paternal effects
Belleannée et al. (2013) [172]	Bovine	Epididymosomes isolated from caput and cauda epididymis; comparative miRNA profiling	miRNA signatures of epididymosomes and epididymal epithelial cells	Distinct miRNA repertoires between caput and cauda epididymosomes and differences from parent epithelial cells, consistent with selective miRNA sorting and intercellular transfer	Provides mechanistic evidence that extracellular vesicles contribute to the remodeling of the sperm-associated miRNA repertoire during epididymal maturation
Gao et al. (2020) [153]	Bovine	Neonatal (3-day) and sexually mature (13-month) testes; RNA sequencing	Testicular miRNA expression and functional analysis of bta-miR-146b	652 miRNAs identified (566 known and 86 novel); 223 were differentially expressed between developmental stages. bta-miR-146b was highly expressed and its overexpression inhibited male germline stem-cell proliferation and promoted apoptosis	Links specific bovine miRNAs with testicular development and germ-cell regulation, providing functional evidence beyond descriptive profiling
Xu et al. (2020) [151]	Cattle, yak and cattleyak	Comparative analysis of spermatogonia and spermatocytes during spermatogenic differentiation	Differential miRNA expression associated with germ-cell differentiation.	147 miRNAs were differentially expressed in cattleyak; 27 were common to both comparisons. Changes in let-7, miR-125, miR-23a and miR-449a were associated with altered spermatogonial differentiation and meiotic progression	Highlights miRNAs as regulators of male germ-cell differentiation and provides a mechanistic context for reproductive phenotypes
Shangguan et al. (2020) [168]	Bull (Bos taurus taurus)	Comparison of fresh and cryopreserved sperm using small-RNA sequencing	Sperm miRNA and mRNA-fragment profiles after cryopreservation	55 miRNAs and 526 mRNA fragments were differentially expressed after cryopreservation. Predicted targets were associated with fertilization, ATP-related processes and apoptosis, including MAPK, autophagy and mitophagy pathways	Demonstrates that cryopreservation can remodel the sperm small-RNA cargo, potentially contributing to post-thaw functional alterations
Keles et al. (2021) [167]	Bos taurus taurus; multiple dairy/beef breeds	18 AI bulls classified as high or low fertility; cryopreserved conventional and sex-sorted sperm	Sperm miRNA abundance and association with non-return rate	85 miRNAs detected. miR-34b-3p and miR-100-5p were most abundant. miR-10a-5p and miR-9-5p differed between fertility groups; several miRNAs correlated with non-return rate after sex sorting	Supports sperm miRNAs as candidate fertility biomarkers, while indicating that associations can depend on semen processing and fertility phenotype
Werry et al. (2022) [156]	Holstein- Friesian	13 bulls classified as high or low fertility according to sire conception rate; semen quality was comparable between groups	Sperm small-RNA sequencing and fertility-associated miRNA analysis	1431 miRNAs detected; five low-count miRNAs were differentially expressed between fertility groups. Several miRNA pairs showed fertility-dependent correlation patterns	Provides evidence for fertility-associated miRNA signatures but also highlights the need for validation because some differential signals had low read counts
Kasimanickam et al. (2022) [163]	Holstein- Friesian	High- and low-fertility bulls; analysis of sperm and seminal plasma miRNAs	Differential miRNA expression and predicted miRNA–mRNA interaction networks	At a ≥5-fold threshold, 32 miRNAs were differentially expressed (20 upregulated and 12 downregulated in high-fertility bulls). Additional candidates emerged using ≥2- and ≥10-fold thresholds	Expands the candidate biomarker repertoire and links sperm/seminal-plasma miRNAs with predicted reproductive molecular networks
Salas-Huetos et al. (2023) [166]	Bovine	29 cryopreserved bovine sperm samples classified according to fertility/non-return rate	Candidate sperm miRNAs, sperm functional variables and fertility potential	Eight of ten candidate miRNAs were detected. miR-138 was significantly lower in subfertile samples and negatively correlated with sperm oxygen consumption; miR-19b, miR-26a and miR-7 correlated with sperm functional variables	Identifies miR-138 and other candidates as potential indicators of sperm functional competence and fertility potential
Zhang et al. (2024) [157]	Dairy cattle	Sperm-borne small-RNA profiling in relation to bull fertility and daughter fertility	Sperm-borne miRNAs and tsRNAs associated with paternal and daughter fertility	Six miRNAs were associated with bull fertility and 35 with daughter fertility. miR-2385-5p and miR-98 showed associations with bull/daughter fertility phenotypes	Strengthens the concept that sperm-borne miRNAs may reflect paternal reproductive performance and potentially contribute to intergenerational effects
Wang et al. (2017) [91]	Bovine	Functional study of sperm-borne miR-449b in bovine SCNT embryos.	Embryonic cleavage, epigenetic reprogramming and apoptosis.	Sperm-borne miR-449b influenced cleavage, epigenetic reprogramming and apoptosis in bovine SCNT embryos.	Provides functional evidence that sperm-borne miRNAs can affect early embryonic development and epigenetic regulation.
Wang et al. (2021) [164]	Bovine	Functional study of sperm-borne miR-202 in bovine embryos.	First cleavage and cytoskeletal regulation in SCNT embryos.	miR-202 was highly enriched in bovine sperm and targeted SEPT7. miR-202 inhibition accelerated first cleavage, whereas miR-202 mimic and SEPT7 knockdown delayed cleavage.	Demonstrates a direct functional role for a sperm-borne miRNA in early embryonic development through cytoskeletal remodeling.
Ďuračka et al. (2026) [165]	Holstein- Friesian	36 bulls; fresh semen miR-31-5p measured in relation to post-thaw sperm freezability.	miR-31-5p, post-thaw motility, viability, mitochondrial membrane potential, apoptosis, ROS and DNA fragmentation.	Higher fresh-sperm miR-31-5p was associated with better post-thaw motility, viability and mitochondrial function and with lower ROS, indicating better freezability.	Identifies miR-31-5p as a candidate molecular marker of sperm resilience to cryopreservation.

AI: artificial insemination; Spz: spermatozoa; sEV: small extracellular vesicles; ROS: reactive oxygen species; miRNA: microRNA; SCNT: somatic cell nuclear transfer; sncRNAs: small non-coding RNAs; tsRNAs: transfer RNA-derived small RNAs.

The sperm miRNA cargo is dynamically shaped by both post-testicular maturation and environmental conditions. During epididymal transit, epididymal epithelial cells secrete EVs, termed epididymosomes, which transfer selected miRNAs and other regulatory small RNAs, including tsRNAs, to maturing spermatozoa [127,172,173]. This RNA remodeling contributes to the acquisition of progressive motility, capacitation, and fertilization competence, while modifying the epigenetic information delivered to the oocyte [126,127,173]. In parallel, environmental challenges, including HS and cryopreservation, can alter the abundance of specific sperm miRNAs involved in oxidative stress, sperm function, and fertilization, linking environmental exposure to impaired male fertility [17,157,163]. Notably, paternal miRNAs have been associated not only with sire fertility but also with the subsequent reproductive performance of female offspring, raising the possibility that environmentally responsive sperm miRNAs contribute to intergenerational epigenetic inheritance [157]. Thus, the mature sperm miRNA profile represents the combined effects of spermatogenesis, epididymal maturation, and environmental exposure, supporting its potential as both a biomarker of male fertility and a mediator of paternal epigenetic inheritance [144].

## 8. Paternal Small RNA Cargo in Fertilization and Early Embryonic Development

The paternal contribution to embryogenesis is no longer viewed as being restricted to the delivery of the haploid genome [133,149]. Accumulating evidence has demonstrated that mature spermatozoa transfer a complex repertoire of sncRNAs, including miRNAs, tsRNAs and piRNAs, which collectively participate in the regulation of maternal transcript clearance, embryonic genome activation and early developmental programming [74,127,133,149,173]. Consequently, sperm-derived RNAs are now regarded as integral components of the paternal epigenome rather than residual products of spermatogenesis [125,173]. The transition from correlation to causality was largely driven by genetic and functional studies targeting the miRNA biogenesis machinery. Conditional disruption of Dicer and Drosha in the mice male germline demonstrated that although spermatozoa deficient in miRNAs retained fertilizing ability, the resulting embryos exhibited defective maternal transcript degradation and impaired developmental competence. This indicated that paternal small RNAs actively regulate preimplantation development [125]. These findings were further supported by mice epididymal RNA-transfer experiments demonstrating that small RNAs acquired during epididymal maturation are required for normal embryonic development [173]. This evidence established sperm RNAs as functional contributors to early embryogenesis, rather than merely residual molecular components of mature spermatozoa. Evidence from cattle indicated that this regulatory paradigm is conserved in livestock species. Embryos generated from high-fertility sires consistently exhibit superior developmental competence together with distinct transcriptomic and miRNA profiles, suggesting that paternal fertility influences embryonic programming well before embryonic genome activation [174,175,176]. It was shown that bovine sperm-derived RNAs actively contribute to embryonic transcriptomic programming before embryonic genome activation, demonstrating that paternal epigenetic contribution extends well beyond genome delivery, although whether this arises directly from miRNAs or a coordinated action of multiple regulatory factors remains unsolved. [84,91].

The paternal small RNA cargo is also highly responsive to physiological and environmental conditions. Age, nutritional status and HS have all been associated with alterations in sperm miRNA composition, supporting the concept that the sperm epigenome is dynamically remodeled throughout adult life [74,169,177]. However, although these molecular alterations are consistently detected, their phenotypic consequences under commercial breeding conditions appear comparatively modest, indicating that additional genetic and environmental factors contribute to offspring performance [161,170,178].

Overall, the available evidence supports a model in which sperm-borne miRNAs function as integral components of a broader paternal epigenetic program, which complements the paternal genome during the earliest stages of embryogenesis [171,179,180]. Rather than functioning as isolated regulators, miRNAs are increasingly recognized as components of an integrated sperm-derived small RNA network comprising tRNA derived small RNAs (tsRNAs), piRNAs, circular RNAs, long non-coding RNAs and RNA-binding proteins, which collectively coordinate maternal transcript degradation, chromatin remodeling and embryonic genome activation [117,127,144,146,157,173]. Nevertheless, despite compelling evidence supporting their role in embryo programming, the causal contribution of individual miRNAs remains incompletely understood because sperm deliver multiple classes of regulatory molecules simultaneously and many miRNAs exhibit functional redundancy [125,181]. Therefore, distinguishing biomarkers of paternal fertility from miRNAs that directly regulate embryonic development remains one of the major challenges in reproductive epigenetics [182]. In fact, the discrimination of miRNAs that actively regulate embryonic development from those that merely reflect sperm physiological status or fertility potential remains elusive [127,157,173]. Future studies combining CRISPR/Cas based genome editing, single-cell multi-omics and quantitative embryo phenotyping will be essential to distinguish these classes of miRNAs [157,182].

## 9. Conclusions

HS is a major environmental challenge to bovine male fertility, impairing spermatogenesis, sperm function, and fertilizing competence through interconnected physiological, cellular, and molecular mechanisms. Current evidence indicates that thermal exposure disrupts redox homeostasis and mitochondrial function, establishing an ROS–mitochondrial axis that can compromise sperm motility, membrane integrity, and cellular survival. HS may also alter CaMKII-dependent signaling and other intracellular pathways involved in sperm activation and acrosomal function. In parallel, changes in sperm RNA cargo, particularly miRNAs, together with alterations in extracellular vesicle-associated miRNAs, suggest that heat exposure can modify the regulatory information delivered by spermatozoa to the oocyte. These molecular changes support the concept that spermatozoa are not only carriers of paternal DNA but also vectors of regulatory molecules with potential consequences for fertilization and early embryonic development. Despite these advances, the functional contribution of specific sperm RNAs and molecular pathways to heat-induced reproductive impairment remains incompletely defined, particularly in cattle.

Future research should prioritize the following three experimental directions: (1) the functional validation of the ROS–mitochondrial and CaMKII signaling pathways in bovine spermatozoa using targeted pathway modulation; (2) the characterization and functional manipulation of specific sperm and extracellular vesicle-associated miRNAs to determine their effects on sperm function, fertilization, and early embryo development; and (3) the integration of sperm and testicular transcriptomics, epigenomics, and reproductive phenotyping across heat-tolerant and heat-sensitive bulls to identify robust molecular biomarkers of thermotolerance. Such approaches will help distinguish conserved mechanisms from cattle-specific responses and establish causal links between thermal exposure, sperm molecular remodeling, and reproductive outcomes. Ultimately, this knowledge may support the development of molecular biomarkers for fertility assessment and breeding strategies aimed at improving the thermal resilience and reproductive efficiency of cattle under increasing climatic stress.

## Figures and Tables

**Table 1:** Landmark studies in miRNA biology.

Year	Study	Species	Main Findings	Current Relevance
2004	Meister and Tuschl [139]	Human	Mechanism of miRNA mediated gene silencing.	Established the canonical miRNA pathway.
2007	Yan et al. [146]	Mouse	miR-34c expression during meiosis.	First evidence of germ-cell-specific miRNA.
2008	Hayashi et al. [147]	Mouse	Dicer-dependent miRNA biogenesis is essential for spermatogenesis.	Established the essential role of miRNAs in germ cell development.
2010	Björk et al. [29]	Mouse	miR-18 regulates HSF2.	Functional regulation of germ cell differentiation.
2011	Wang et al. [148]	Human	Seminal plasma miRNAs associated with asthenozoospermia.	First biomarker panel for male infertility.
2011	Krawetz et al. [149]	Human	Sperm miRNAs contribute to early embryogenesis.	Introduced paternal epigenetic contribution.
2012	Govindaraju et al. [116]	Bull	Dynamic miRNA profile of bull spermatozoa.	Established sperm miRNAs as potential biomarkers of semen quality and fertility.

## Data Availability

No new data were created or analyzed in this study. Data sharing is not applicable.
